# Prediction of metabolism and solubility of tablet-form drugs according to the biopharmaceutical drug disposition classification system

**DOI:** 10.5599/admet.2945

**Published:** 2025-09-17

**Authors:** Yaroslava Pushkarova, Galina Zaitseva, Kaouthar Bouaalam

**Affiliations:** Department of Analytical, Physical and Colloid Chemistry, Bogomolets National Medical University, Kyiv, 01601, Ukraine

**Keywords:** Molecular descriptors, probabilistic neural network, chemometrics, drug classification

## Abstract

**Background and purpose:**

Prediction of metabolism and solubility of tablet-form drugs is essential in pharmaceutical development, impacting drug efficacy, safety and formulation strategies. This study aimed to develop predictive models for classifying drugs according to metabolism and solubility within the Biopharmaceutical Drug Disposition Classification System.

**Experimental approach:**

A dataset of 220 tablet-form drugs characterized by eleven molecular descriptors was analysed. The Kruskal–Wallis test identified relevant descriptors for metabolism (extensive vs. poor) and solubility (high vs. low) classifications. Probabilistic Neural Networks were employed for predictive modelling, with model parameters optimized to enhance accuracy.

**Key results:**

Six molecular descriptors (hydrogen bond acidity, logarithm of the partition coefficient, distribution coefficient, hydrogen bond acceptor count, molecular weight and polar surface area) predicted metabolism class with 97 % accuracy. For solubility classification, five descriptors (dipolarity/polarizability, logarithm of the partition coefficient, distribution coefficient, hydrogen bond donor count and molecular weight) achieved 88 % accuracy. Removal of key descriptors significantly reduced model performance, confirming their importance.

**Conclusion:**

The developed models demonstrate robust predictive capability for drug metabolism and solubility classes as defined by the Biopharmaceutical Drug Disposition Classification System, supporting early-stage drug screening based solely on molecular structure. The lower accuracy observed for solubility prediction reflects its complex and multifactorial nature, highlighting the need for further refinement of molecular descriptors. These findings advance the field by providing effective computational tools to predict key biopharmaceutical properties, potentially accelerating the drug development process.

## Introduction

Predicting the metabolism and solubility of pharmaceutical compounds is a critical aspect of drug development and personalized medicine. Metabolism primarily affects how drugs are processed in the body, while solubility determines the extent of drug absorption. These properties influence bioavailability, therapeutic efficacy, dosing strategies, and the risk of adverse effects. Accurate prediction of whether a drug exhibits poor or extensive metabolism and solubility is essential for optimizing pharmacokinetic profiles and ensuring patient safety [1-3].

The Biopharmaceutical Drug Disposition Classification System (BDDCS), introduced by Wu and Benet in 2005 [4], is a well-established framework that categorizes drugs based on their solubility and extent of metabolism, thereby providing enhanced predictive insight into their absorption, distribution, metabolism and excretion properties. Unlike the earlier Biopharmaceutics Classification System, which focused primarily on solubility and permeability, BDDCS integrates metabolic data to better predict drug disposition and potential formulation challenges [5].

BDDCS classifies drugs into four categories based on their solubility and extent of metabolism. Class 1 drugs exhibit high solubility and extensive metabolism, whereas Class 2 drugs have low solubility but still undergo extensive metabolism. Class 3 includes drugs with high solubility but poor metabolism, and Class 4 comprises drugs characterized by both low solubility and poor metabolism. This classification aids in anticipating bioavailability issues, understanding transporter effects, and predicting drug-drug interactions, while also providing insights relevant to food-drug interaction potential [6,7].

Given the importance of accurately predicting these properties to enhance drug development and improve patient outcomes, this study aims to develop predictive models for the metabolism and solubility of tablet-form drugs based on the principles of the BDDCS, utilizing molecular descriptors and chemometric methods.

## Experimental

### Dataset of tablet-form drugs and molecular descriptors

The studied dataset includes 220 tablet-form drugs characterized by eleven molecular descriptors: hydrogen bond acidity (*A*), hydrogen bond basicity (*B*), dipolarity/polarizability (*S*), excess molar refraction (*E*), logarithm of the partition coefficient (log *P*), distribution coefficient (log *D*), rotatable bond count (RotB), hydrogen bond acceptor count (HBA), hydrogen bond donor count (HBD), molecular weight (MW), and polar surface area (PSA). Values for these descriptors were obtained from the PubChem database (https://pubchem.ncbi.nlm.nih.gov/) and [8]. log *P* represents the partition coefficient of the neutral molecule, independent of pH, reflecting its intrinsic hydrophobicity. In contrast, log *D* accounts for the distribution between ionized and unionized species at physiological pH 7.4, making it directly relevant for drug absorption and distribution processes. In this study, the prediction of metabolism and solubility of tablet-form drugs according to the Biopharmaceutical Drug Disposition Classification System is understood as classification into metabolic and solubility classes: metabolism is categorized as extensive or poor (corresponding to classes 1 and 2), while solubility is classified as high or low (also corresponding to classes 1 and 2).

### Used methods and algorithms

The present study employed the Kruskal–Wallis test, Probabilistic Neural Network (PNN), and correlation analysis as primary research methods. All computational analyses were performed using MATLAB R2024b software package (https://www.mathworks.com/).

The Kruskal–Wallis test is a non-parametric method used to determine whether there are statistically significant differences between the distributions of three or more independent groups. This test is based on the ranks of parameter values rather than their raw numerical values, allowing analysis of non-normally distributed data. It compares the calculated chi-square statistic to the critical value from the chi-square distribution table. If the calculated value exceeds the critical value, this indicates statistically significant differences in the tested parameter across groups, implying that the parameter influences group classification. Otherwise, no significant differences are found among groups. In this study, the Kruskal–Wallis test was specifically used to identify which molecular descriptors significantly affect the classification of tablet-form drugs according to their metabolism (extensive vs. poor) and solubility (high vs. low) within the Biopharmaceutical Drug Disposition Classification System [9,10].

To ensure the appropriateness of nonparametric analysis, the distribution of all molecular descriptors was first assessed. Normality was evaluated using the Lilliefors test, complemented by skewness and kurtosis statistics [11,12]. The Lilliefors test is a modification of the Kolmogorov–Smirnov test suitable when the mean and standard deviation are estimated from the sample rather than known a priori. This test determines whether a variable deviates from a normal distribution, returning a significance value (*p*-value) and a binary indicator *h* (1 if non-normal, 0 if normal). The results (Table 1) show that only two descriptors (logarithm of the partition coefficient and distribution coefficient) followed a normal distribution (*p* ≥ 0.05), while the remaining nine descriptors significantly deviated from normality (*p* < 0.05). Skewness and kurtosis values further confirm asymmetry and heavy tails in the distributions of non-normal descriptors. These findings support the use of the nonparametric Kruskal–Wallis test to identify descriptors that significantly influence group classification.

**Table 1.** Normality assessment of molecular descriptors

Descriptor	Skewness	Kurtosis	Lilliefors *p*	Lilliefors *h*
Hydrogen bond acidity	0.95	3.89	0.0010	1
Hydrogen bond basicity	1.54	6.03	0.0010	1
Dipolarity/polarizability	0.50	3.30	0.0048	1
Excess molar refraction	3.18	26.74	0.0010	1
Logarithm of the partition coefficient	0.037	2.49	0.50	0
Distribution coefficient	0.15	3.71	0.50	0
Rotatable bond count	1.26	6.60	0.0010	1
Hydrogen bond acceptor count	2.38	15.02	0.0010	1
Hydrogen bond donor count	0.95	3.77	0.0010	1
Molecular weight	1.73	8.87	0.0010	1
Polar surface area	0.64	3.13	0.0024	1

A Probabilistic Neural Network is a feedforward neural network used primarily for classification. Based on Bayesian theory, it employs kernel-based probability estimation to calculate the likelihood of an input belonging to each class. Its architecture consists of four layers: input, pattern (with neurons representing training samples), summation (aggregating class probabilities), and output (selecting the class with the highest probability). PNNs offer fast training, high accuracy, and robustness to noise, but can be memory-intensive and slower during prediction on large datasets due to explicit storage of training samples [13]. A key parameter in PNN is the spread, which controls the smoothness of the decision boundaries by influencing the width of the kernel functions. Selecting an appropriate spread value is crucial for balancing generalization and overfitting. We analysed the effect of the spread parameter on classification performance by testing values ranging from 0.1 to 1.0 to identify the interval where the model achieves optimal results.

Since the Probabilistic Neural Network is a supervised learning model, the dataset of 220 tablet-form drugs characterized by eleven molecular descriptors was randomly divided into a training subset (85 %) used for model training and a testing subset (15 %) reserved for independent evaluation of predictive performance.

It is important to note that in a Probabilistic Neural Network, each training sample contributes a kernel function in the pattern layer. Specifically, for every compound in the training set, a neuron is created in this layer, and its associated kernel (typically Gaussian) represents the probability density centred at that sample. During classification, the network evaluates how closely an input matches these kernels across all classes.

In our dataset, all training samples were correctly classified for spread values ranging from 0.1 to 1.0, as each sample essentially «remembers» its own position in feature space. Misclassifications occurred only for unseen compounds in the independent test set, where inputs may lie between kernels or in sparsely populated regions. This behaviour highlights the network’s ability to perfectly memorize the training set while still generalizing effectively to new data.

Together, the Kruskal–Wallis test and Probabilistic Neural Network represent valuable tools in chemometric and biomedical data analysis, especially for assessing group differences and developing predictive models based on biological or chemical descriptors [14].

## Results and discussion

### Prediction of BDDCS class according to metabolism (extensive or poor metabolism)

The Kruskal–Wallis test was applied to 11 molecular descriptors of 220 tablet-form drugs, and the results are summarized in Table 2. The critical *χ*^2^ value at a 5 % significance level with 1 degree of freedom is 3.84 [15].

**Table 2.** Results of the Kruskal–Wallis test for molecular descriptors influencing metabolism classification

	Descriptor										
	*A*	*B*	*S*	*E*	log *P*	log *D*	RotB	HBA	HBD	MW	PSA
*χ* ^2^	55.98	16.65	0.25	0.10	66.70	64.26	0.010	25.41	51.13	4.96	39.62

The results indicate that dipolarity/polarizability, excess molar refraction and rotatable bond count have negligible influence on the classification/prediction of BDDCS class according to metabolism (extensive vs. poor metabolism).

To assess multicollinearity between descriptors, correlation coefficients were calculated for each pair of parameters. The results are presented in Table 3. Hydrogen bond acidity and hydrogen bond donor count are strongly correlated, as are hydrogen bond basicity and hydrogen bond acceptor count. From each correlated pair, the parameter with the higher *χ*^2^ value (i.e., the greater influence on metabolism classification) was retained – hydrogen bond acidity and hydrogen bond acceptor count.

**Table 3.** Correlation coefficients between molecular descriptors

Descriptor	*A*	*B*	*S*	*E*	log *P*	log *D*	RotB	HBA	HBD	MW	PSA
A	1.00	0.54	0.29	-0.06	-0.43	-0.41	0.16	0.46	**0.89**	0.20	0.77
B	0.54	1.00	0.58	-0.05	-0.21	-0.16	0.50	**0.85**	0.65	0.74	0.82
S	0.29	0.58	1.00	0.00	0.07	0.08	0.25	0.50	0.23	0.60	0.51
E	-0.06	-0.05	0.00	1.00	0.07	0.03	0.01	-0.01	-0.08	-0.04	-0.06
Log *P*	-0.43	-0.21	0.07	0.07	1.00	0.79	0.16	-0.22	-0.46	0.37	-0.42
Log *D*	-0.41	-0.16	0.08	0.03	0.79	1.00	0.18	-0.09	-0.40	0.41	-0.29
RotB	0.16	0.50	0.25	0.01	0.16	0.18	1.00	0.47	0.24	0.61	0.38
HBA	0.46	**0.85**	0.50	-0.01	-0.22	-0.09	0.47	1.00	0.59	0.67	0.80
HBD	**0.89**	0.65	0.23	-0.08	-0.46	-0.40	0.24	0.59	1.00	0.26	0.81
MW	0.20	0.74	0.60	-0.04	0.37	0.41	0.61	0.67	0.26	1.00	0.50
PSA	0.77	0.82	0.51	-0.06	-0.42	-0.29	0.38	0.80	0.81	0.50	1.00

Thus, for the classification/prediction of metabolism (extensive vs. poor), six molecular descriptors are recommended: hydrogen bond acidity, logarithm of the partition coefficient, distribution coefficient, hydrogen bond acceptor count, molecular weight and polar surface area.

Based on the selected molecular descriptors identified through the Kruskal–Wallis test and correlation analysis, a Probabilistic Neural Network model was constructed to classify tablet-form drugs according to their metabolism profiles. To optimize the model’s predictive performance, different spread parameter values ranging from 0.1 to 1.0 were evaluated. The classification results for the independent testing subset are summarized in Table 4, where misclassified drugs are highlighted in bold. The lowest misclassification rate was observed for spread values between 0.8 and 1.0, with only azathioprine misclassified, yielding an overall accuracy of 97 %.

**Table 4.** Results of metabolism prediction using a Probabilistic Neural Network (1 – extensive metabolism, 2 – poor metabolism)

Drug	Correct BDDCS class	PNN spread values									
		0.1	0.2	0.3	0.4	0.5	0.6	0.7	0.8	0.9	1.0
		Predicted class									
Acetohexamide	1	1	1	1	1	1	1	1	1	1	1
Aliskiren	1	1	1	1	1	1	1	1	1	1	1
Alosetron	1	1	1	1	1	1	1	1	1	1	1
Alprazolam	1	1	1	1	1	1	1	1	1	1	1
Ambrisentan	1	1	1	1	1	1	1	1	1	1	1
Aminophenazone	1	1	1	1	1	1	1	1	1	1	1
Amlodipine	1	1	1	1	1	1	1	1	1	1	1
Amoxapine	1	1	1	1	1	1	1	1	1	1	1
Anastrozole	1	1	1	1	1	1	1	1	1	1	1
Asenapine	1	1	1	1	1	1	1	1	1	1	1
Azathioprine	1	1	1	1	**2**	**2**	**2**	**2**	**2**	**2**	**2**
Bambuterol	1	1	1	1	1	1	1	1	1	1	1
Melphalan	1	1	1	1	1	1	1	1	1	1	1
Omeprazole	1	1	1	1	1	1	1	1	1	1	1
Prochlorperazine	1	1	1	1	1	1	1	1	1	1	1
Thioridazine	1	1	1	1	1	1	1	1	1	1	1
Triamcinolone acetonide	1	1	1	1	1	1	1	1	1	1	1
Aminoglutethimide	1	1	1	1	1	1	1	1	1	1	1
Aripiprazole	1	1	1	1	1	1	1	1	1	1	1
Armodafinil	1	1	1	1	1	1	1	1	1	1	1
Astemizole	1	1	1	1	1	1	1	1	1	1	1
Bevantolol	1	1	1	1	1	1	1	1	1	1	1
Bicalutamide	1	1	1	1	1	1	1	1	1	1	1
Bosentan	1	1	1	1	1	1	1	1	1	1	1
Albuterol	2	**1**	2	2	2	2	2	2	2	2	2
Amiloride	2	**1**	**1**	**1**	**1**	**1**	**1**	**1**	2	2	2
Amoxicillin	2	**1**	**1**	**1**	**1**	2	2	2	2	2	2
Atenolol	2	1	2	2	2	2	2	2	2	2	2
Baclofen	2	**1**	**1**	**1**	2	2	2	2	2	2	2
Cadralazine	2	**1**	**1**	**1**	**1**	2	2	2	2	2	2
Captopril	2	**1**	**1**	**1**	2	2	2	2	2	2	2
Acyclovir	2	**1**	**1**	**1**	2	2	2	2	2	2	2
Cefpodoxime	2	**1**	**1**	**1**	**1**	**1**	**1**	2	2	2	2

We also examined the effect of the molecular weight descriptor on the classification/prediction of BDDCS metabolism classes (extensive vs. poor), since its *χ*^2^ value was notably lower than those of the other descriptors (see Table 2). Removing the molecular weight descriptor from the parameter set led to a significant increase in the proportion of misclassified drugs, reducing the overall prediction accuracy from 97 to 85 % across spread values between 0.8 and 1.0. Specifically, eight compounds were misclassified across different spread values, including aliskiren, azathioprine, melphalan, armodafinil, albuterol, amiloride, captopril and acyclovir. These results indicate that the molecular weight descriptor is informative and necessary for accurate classification.

### Prediction of BDDCS class according to solubility (high or low solubility)

Similarly, we analysed the influence of molecular descriptors on the classification/prediction of BDDCS solubility classes (high *vs.* low). The Kruskal-Wallis test was performed for 11 molecular descriptors. The results of the test are presented in Table 5. The critical *χ*^2^ value at a significance level of 5 % with 1 degree of freedom is 3.84 [13].

**Table 5.** Results of the Kruskal–Wallis test for molecular descriptors influencing solubility classification

	Descriptor										
	*A*	*B*	*S*	*E*	Log *P*	Log *D*	RotB	HBA	HBD	MW	PSA
*χ* ^2^	1.19	0.00	18.69	1.71	18.71	38.81	0.74	0.21	5.44	17.57	0.01

For the classification/prediction of solubility (high or low), we recommend five molecular descriptors: dipolarity/polarizability, logarithm of the partition coefficient, distribution coefficient, hydrogen bond donor count and molecular weight. No multicollinearity was observed among these parameters (see Table 3).

Based on the selected molecular descriptors identified through the Kruskal–Wallis test, a Probabilistic Neural Network model was constructed to classify tablet-form drugs according to their solubility profiles. To optimize the model’s predictive performance, different spread parameter values ranging from 0.1 to 1.0 were evaluated. The classification results for the testing subset are summarized in Table 6, with misclassified drugs highlighted in bold. The lowest misclassification rate was observed for spread values between 0.7 and 1.0, where four drugs – bambuterol, aminoglutethimide, aripiprazole and atenolol – were misclassified, resulting in an overall prediction accuracy of 88 %.

**Table 6.** Prediction results for solubility using a Probabilistic Neural Network (1 – high solubility, 2 – low solubility)

Drug	Correct BDDCS class	PNN spread values									
		0.1	0.2	0.3	0.4	0.5	0.6	0.7	0.8	0.9	1.0
		Predicted class									
Acetohexamide	1	1	1	1	1	1	1	1	1	1	1
Aliskiren	1	1	1	1	1	1	1	1	1	1	1
Alosetron	1	1	1	1	1	1	1	1	1	1	1
Alprazolam	1	1	1	1	1	1	1	1	1	1	1
Ambrisentan	1	1	1	1	1	1	1	1	1	1	1
Aminophenazone	1	1	1	1	1	1	1	1	1	1	1
Amlodipine	1	1	1	1	1	1	1	1	1	1	1
Amoxapine	1	1	1	1	1	1	1	1	1	1	1
Anastrozole	1	1	1	1	1	1	1	1	1	1	1
Asenapine	2	1	2	2	2	2	2	2	2	2	2
Azathioprine	2	2	2	2	2	2	2	2	2	2	2
Bambuterol	2	**1**	**1**	**1**	**1**	**1**	**1**	**1**	**1**	**1**	**1**
Melphalan	2	2	2	2	2	2	2	2	2	2	2
Omeprazole	2	2	2	2	2	2	2	2	2	2	2
Prochlorperazine	2	2	2	2	2	2	2	2	2	2	2
Thioridazine	2	2	2	2	2	2	2	2	2	2	2
Triamcinolone acetonide	2	**1**	2	2	2	2	2	2	2	2	2
Aminoglutethimide	2	**1**	**1**	**1**	**1**	**1**	**1**	**1**	**1**	**1**	**1**
Aripiprazole	1	1	**2**	**2**	**2**	**2**	**2**	**2**	**2**	**2**	**2**
Armodafinil	1	1	1	1	1	1	1	1	1	1	1
Astemizole	1	1	1	1	1	1	1	1	1	1	1
Bevantolol	1	1	1	1	1	1	1	1	1	1	1
Bicalutamide	1	1	1	1	1	1	1	1	1	1	1
Bosentan	1	1	1	1	1	1	1	1	1	1	1
Albuterol	1	1	1	1	1	1	1	1	1	1	1
Amiloride	1	1	1	1	1	1	1	1	1	1	1
Amoxicillin	1	1	1	1	1	1	1	1	1	1	1
Atenolol	1	**2**	**2**	**2**	**2**	**2**	**2**	**2**	**2**	**2**	**2**
Baclofen	2	**1**	**1**	**1**	**1**	**1**	**1**	2	2	2	2
Cadralazine	2	1	2	2	2	2	2	2	2	2	2
Captopril	2	2	2	2	2	2	2	2	2	2	2
Acyclovir	2	2	2	2	2	2	2	2	2	2	2
Cefpodoxime	2	**1**	**1**	**1**	**1**	**1**	2	2	2	2	2

The results demonstrate the effectiveness of the proposed models in predicting the metabolism and solubility categories of tablet-form drugs, offering a valuable tool for early-stage drug development. The high accuracy achieved underscores the critical importance of selecting appropriate molecular descriptors and optimizing model parameters to ensure robust and reliable predictions.

The identified molecular descriptors highlight both shared and unique physicochemical properties influencing metabolism and solubility classification. Notably, the logarithm of the partition coefficient, distribution coefficient and molecular weight play central roles in predicting both parameters, reflecting their fundamental influence on drug behaviour. In contrast, descriptors such as hydrogen bond acidity, hydrogen bond acceptor count and polar surface area are specifically relevant to metabolism, whereas dipolarity/polarizability and hydrogen bond donor count are more critical for solubility. This distinction highlights the distinct biochemical and physicochemical mechanisms that govern these two crucial biopharmaceutical properties.

The observed difference in predictive accuracy between metabolism and solubility classifications can be attributed to the inherently multifactorial nature of solubility. While metabolism is largely governed by specific enzymatic processes and thus may be more directly associated with certain molecular features, solubility is influenced by a broader spectrum of physicochemical and structural factors. These include molecular interactions with solvents, crystalline forms, polymorphism, and dynamic conformational changes, many of which are not fully captured by the selected molecular descriptors. This limitation suggests that improving solubility prediction models may require the incorporation of additional descriptors or alternative data sources that better represent these complex influences. For instance, integrating descriptors related to crystal lattice energy, hydration shell characteristics, or employing three-dimensional molecular descriptors could enhance model performance.

## Conclusions

Predictive models based on a Probabilistic Neural Network were successfully developed for the classification of tablet-form drugs according to their metabolism and solubility profiles within the framework of the Biopharmaceutical Drug Disposition Classification System. The Probabilistic Neural Network was evaluated across a range of spread values from 0.1 to 1.0. For metabolism classification, the lowest proportion of misclassified drugs occurred at spread values between 0.8 and 1.0, while for solubility classification, this range was slightly broader, from 0.7 to 1.0.

In metabolism prediction (extensive vs. poor), six key molecular descriptors – hydrogen bond acidity, logarithm of the partition coefficient, distribution coefficient, hydrogen bond acceptor count, molecular weight and polar surface area – were identified, yielding a prediction accuracy of 97 %.

In solubility classification (high vs. low), five molecular descriptors – dipolarity/polarizability, logarithm of the partition coefficient, distribution coefficient, hydrogen bond donor count and molecular weight – were applied, achieving an accuracy of 88 %.

The lower accuracy for solubility prediction can be explained by the multifactorial nature of solubility, which depends on additional physicochemical and structural parameters not fully represented by the selected descriptors.

The use of Probabilistic Neural Network enabled effective classification by capturing nonlinear relationships between molecular descriptors and biopharmaceutical properties. The developed models demonstrate high predictive potential and may serve as a reliable tool for early-stage drug screening, allowing for efficient assessment of metabolism and solubility based solely on molecular structure.
